# The Biology of Placebo and Nocebo Effects on Experimental and Chronic Pain: State of the Art

**DOI:** 10.3390/jcm12124113

**Published:** 2023-06-18

**Authors:** Giacomo Rossettini, Francesco Campaci, Joel Bialosky, Eva Huysmans, Lene Vase, Elisa Carlino

**Affiliations:** 1 School of physiotherapy, University of Verona, 37129 Verona, Italy; 2 Department of Neuroscience “Rita Levi Montalcini”, University of Turin, 10124 Turin, Italy; 3 Department of Physical Therapy, University of Florida, Gainesville, FL 32611, USA; 4 Clinical Research Center, Brooks Rehabilitation, Jacksonville, FL 32211, USA; 5 Pain in Motion Research Group (PAIN), Department of Physiotherapy, Human Physiology and Anatomy, Faculty of Physical Education & Physiotherapy, Vrije Universiteit Brussel, Laarbeeklaan 103, 1090 Brussels, Belgium; 6 Department of Physical Medicine and Physiotherapy, Universitair Ziekenhuis Brussel, Laarbeeklaan 101, 1090 Brussels, Belgium; 7 Department of Psychology and Behavioural Sciences, School of Business and Social Sciences, Aarhus University, 8000 Aarhus, Denmark

**Keywords:** placebo effect, nocebo effect, expectation, conditioning, pain, contextual factor

## Abstract

(1) Background: In recent years, placebo and nocebo effects have been extensively documented in different medical conditions, including pain. The scientific literature has provided strong evidence of how the psychosocial context accompanying the treatment administration can influence the therapeutic outcome positively (placebo effects) or negatively (nocebo effects). (2) Methods: This state-of-the-art paper aims to provide an updated overview of placebo and nocebo effects on pain. (3) Results: The most common study designs, the psychological mechanisms, and neurobiological/genetic determinants of these phenomena are discussed, focusing on the differences between positive and negative context effects on pain in experimental settings on healthy volunteers and in clinical settings on chronic pain patients. Finally, the last section describes the implications for clinical and research practice to maximize the medical and scientific routine and correctly interpret the results of research studies on placebo and nocebo effects. (4) Conclusions: While studies on healthy participants seem consistent and provide a clear picture of how the brain reacts to the context, there are no unique results of the occurrence and magnitude of placebo and nocebo effects in chronic pain patients, mainly due to the heterogeneity of pain. This opens up the need for future studies on the topic.

## 1. Introduction

In recent years, placebo and nocebo effects have strongly influenced pain studies, which fostered the interest in this topic and encouraged debate among scholars, researchers, and clinicians worldwide [1,2,3].

From their earliest days, placebos have been identified as inert substances (e.g., sugar pills, saline injections) used in clinical trials to control the efficacy of new treatments [3]. Today, neuroscientists and clinicians recognize that placebos are more than inert substances, introducing the concept of “context surrounding a treatment” [4]. Accordingly, placebo and nocebo effects are now defined as, respectively, the positive or negative effects due to the administration of a treatment (be it real or simulated) in a therapeutic context [5]. The context that triggers these effects comprises symbols, rituals, and cues (e.g., provider’s words, patient’s expectations and previous experiences, physical aspects of the treatment) that accompany patients during their healthcare experiences [6,7]. In the field of pain, the administration of an inert treatment in a positive context can induce a reduction of pain (as reported by subjective pain reports) as well as a modulation of specific brain circuits involved in pain processing. On the contrary, when an inert treatment is administered in a negative context, participants/patients can experience pain exacerbation as well as increased activation of pain-related brain regions [8]. Similarly, it has been documented that administering treatments without a proper context (e.g., when patients are unaware that a medication/drug has been delivered) strongly reduced the efficacy of the medication [9].

Thus, from this perspective, analyzing how the therapeutic context can influence treatment efficacy represents an opportunity for both clinicians and researchers. This state-of-the-art paper aims to provide an updated overview of placebo and nocebo studies on pain, showing how treatments (active or inert) administered in positive or negative contexts trigger different outcomes. Thus, this paper will serve to help clinicians to be more aware of the use of context in their medical routine. Moreover, it will serve to help researchers to build upon the best evidence for designing future trials and implementing new studies to increase our knowledge on the biological determinants of placebo and nocebo effects on pain. The first section provides the reader with a solid background of the mechanisms and the neurobiological determinants of placebo and nocebo effects on pain. The second section describes the future implications for clinical practice to maximize the medical routine. Moreover, implications for research are discussed to help researchers design future trials and develop new innovative studies on pain.

This state-of-the-art paper has been prepared and developed following methodological guidelines for narrative reviews (Table 1) [10]. The articles included in this state-of-the-art overview needed to (1) be scientific works (experimental studies, systematic or narrative reviews (including meta-analyses), or RTCs) published in peer-reviewed journals; (2) be primarily focused on the analgesia/hyperalgesia manifestation of placebo/nocebo effects and/or on the psychological and neurobiological mechanisms involved; (3) provide significant data for a comprehensive, descriptive, and state-of-the-art overview; and (4) provide a detailed description of the methodological approaches used (only in the case of experimental articles). Additionally, the articles presented in Section 3 needed to focus on chronic pain conditions, specifically. Overall, 80 experimental studies and RCTs on placebo and nocebo effects on healthy volunteers and chronic pain patients have been reviewed. Study characteristics of these experimental studies are summarized in Table A1 (Appendix A), including the sample size, population involved, pain type or pain type induction, investigated outcome, objective measures, and level of significance reported by the authors. Furthermore, 31 reviews and 24 meta-analyses and systematic reviews have been included in order to provide a clear and broad overview of the literature concerning placebo/nocebo phenomena in healthy volunteers and chronic pain patients.

## 2. State of the Art

### 2.1. Experimental Approaches to Study Placebo and Nocebo Effects

Placebo and nocebo effects on pain have been extensively studied using experimental research designs [6,11,12,13,14,15]. Different approaches have been used to trigger pain amelioration or exacerbation: the two most common procedures are (1) the use of positive or negative expectations and (2) the use of conditioning approaches [11,12]. In the first case, inert treatments are administered along with verbal information that a real treatment is delivered: using this approach, participants or patients are made to believe that a treatment is administered and a positive or negative effect is expected [16,17,18,19]. In the second case, using conditioning protocols, a real treatment is administered for different trials and subsequently replaced by an inert treatment: using this approach, participants or patients experience a positive effect when the active treatment is administered, and they expect the same effect when the inert treatment is delivered unbeknownst to them [20,21,22]. Studies in healthy volunteers showed that conditioning protocols produce more robust [23,24] and long-lasting placebo effects that cannot be attributable to carryover effects of the active treatment. On the contrary, nocebos seem to result in a great worsening of pain even without a conditioning procedure [25]. Interestingly, the conditioned placebo effect seems to be transferable from one modality (analgesia conditioning) to another (motor performance) [26].

Besides expectation and conditioning studies, context effects have been extensively documented using the so-called “open-hidden” design, in which participants or patients receive a real analgesic drug in two different conditions: in the open condition, they are aware that the drug is administered (presence of the context), in the hidden one, they are unaware of receiving it (absence of the context) [27]. Studies consistently find pain relieving medication of established effectiveness to be significantly more effective when administered in an open fashion as compared to when individuals are unaware of receiving the medication [28]. Thus, the difference between the two conditions shows how exposure to a context influences the effectiveness of a treatment which is in fact proven to be active. Recently, another approach has been used is the open–label nondeceptive approach, whereby participants are informed that an inert treatment will be administered, and that this treatment can be effective [11,12,29,30]. These two approaches (open–hidden and open–label) offer the possibility to study placebo effects in clinical settings without the ethical controversies of deception: indeed, in the first case, a real drug is administered, and the effect of the context is studied without using an inert treatment. In the second case, the use of a placebo is fully disclosed.

### 2.2. Neurobiology

Over the last few decades, different studies and projects have been conducted, using different approaches ranging from pharmacology to neuroimaging [31,32,33], to describe the brain circuitry and neurotransmitter systems that trigger or block placebo and nocebo effects. The study of the neurobiological determinants of these phenomena is crucial for different reasons: (1) it provides solid knowledge of the objective effects of the context on our brain, (2) it demonstrates that placebo/nocebo and drugs share common biochemical pathways and activate the same receptor pathways, which suggests possible interference between the context and rituals that surround a treatment on the one hand and pharmacological agents on the other. Major studies on healthy participants exposed to experimental pain will be discussed in the next sections. Subsequently, a focus on patients with chronic pain will be presented.

#### 2.2.1. Pharmacological Evidence

**Figure 1:**
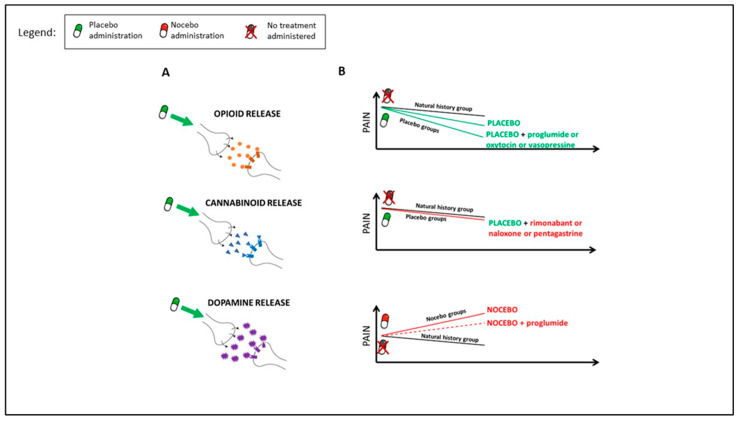
Pharmacological evidences. As reported by different pharmacological studies, placebo administration activates endogenous opioid, cannabinoid, and dopamine systems (**A**). Participants in the placebo groups experienced analgesic effects, namely pain reduction, compared to participants that received no treatments (natural history group). This analgesic effect is enhanced by proglumide, oxytocin, and vasopressine ((**B**), upper graph) while it is disrupted by rimonabant, naloxone, and pentagastrin ((**B**), middle graph). Nocebo effects exacerbate pain perception compared no treatment groups (natural history group). This effect is partially reversed by CCK antagonist proglumide ((**B**), bottom graph).

Pharmacological studies demonstrated that inert treatments activate the endogenous opioid and endocannabinoid systems (Figure 1A). In these studies, conducted on healthy volunteers, a conditioning protocol was induced, in which opioids (e.g., morphine) or cannabinoids (e.g., ketorolac) were administered and subsequently replaced by a placebo. After morphine administration, µ-opioid antagonists (e.g., naloxone) block placebo analgesia [20,34,35]. The same effect has been discovered using CB1-antagonist (e.g., rimonabant) after cannabinoid administration [36]. Interestingly, naloxone has also been seen to block open–label nondeceptive placebo analgesia, indicating that the same mechanisms may mediate nondeceptive and deceptive placebo analgesia [37]. Indirect confirmations of the involvement of the opioid system have been reported investigating the role of cholecystokinin (CCK), an anti-opioid peptide, and in particular, the role of CCK antagonists (e.g., proglumide) and CCK agonists (e.g., pentagastrin). Proglumide enhances placebo analgesic effects while pentagastrin disrupts them [38,39,40,41,42]. Furthermore, nocebo hyperalgesia seems to be modulated by the activation of the opioid system, as CCK antagonists can reverse it [38]. Scott et al. (2008) [43] found a deactivation of the μ-opioid receptor system during nocebo hyperalgesia (Figure 1B).

Beside opioid and cannabinoid systems, the dopamine system has been explored in this context [32,33]. Some studies indicate that dopamine may be involved in placebo analgesia influencing the activity of pain-related areas, such as the thalamus, insula, anterior cingulate cortex [44,45], and the ventrolateral prefrontal cortex [46]. These data are controversial. Indeed, it is likely that dopamine may not be fundamental for placebo analgesia itself [47,48], but it may be more generally involved in placebo responsiveness [46,49]. In particular, dopamine may affect patients’ expectations and desire for improvement [47] and the recalled efficacy of a placebo [46].

Other neurotransmitters, e.g., oxytocin and vasopressin, may be involved in expectancy-induced analgesia [50,51]. Interestingly, the administration of vasopressin has been observed to be associated with increased placebo analgesia, but the effect was restricted to women [50]. The hypothesis behind the involvement of these neurotransmitters takes into account their role in social behavior [52,53], but the results are still preliminary, as other studies do not support the facilitating effect of oxytocin on placebo analgesia [50]. Finally, placebos and nocebos modulate the synthesis of prostaglandins, being important targets of analgesic drugs [54], and the plasma level of pro-inflammatory cytokine (IL-18) during pain experience [55]. It is crucial to consider that the mechanisms addressed above were studied in healthy volunteers exposed to experimental pain protocols. As will be discussed below, fewer studies investigated placebo and nocebo effects in patients with chronic pain, and it has been suggested that the knowledge derived from studies on healthy volunteers may not be entirely transferrable to chronic pain populations [56].

#### 2.2.2. Neuroimaging Studies

Neuroimaging studies have provided crucial insights into how exposure to a context can positively change pain perception at different temporal phases and high and low levels of the central nervous system [57,58,59,60,61,62,63,64,65].

##### Temporal Aspects

**Figure 2:**
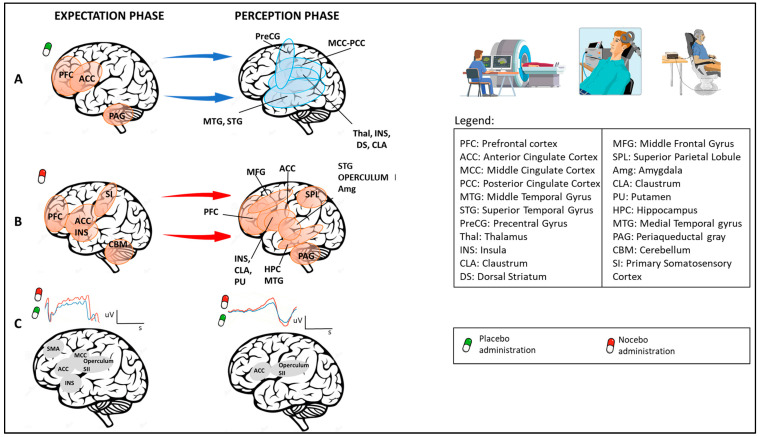
Neuroimaging studies: temporal aspects (expectation and perception phases) related to brain area activity after placebo or nocebo administration. As reported by different neuroimaging studies, expectations of pain relief, triggered by placebos, activate brain areas such as PFC, ACC, and PAG (P1); in the perception phase, deactivation has been found in different brain regions, including MCC, PCC, MTG, STG, PreCG, Thal, INS, CLA, and DS (**A**). On the contrary, expectations of pain worsening, triggered by nocebos, enhance activity in brain regions that include PFC, ACC, INS, SI, and CBM; in the perception phase, increased activity in PFC, ACC, MFG, INS, CLA, PU, HPC, MTG, SPL, STG, OPERCULUM, and INS has been found (**B**). Electroencephalographic (EEG) studies report that placebos and nocebos change EEG brain activity. In particular, the expectation of receiving no painful or painful stimuli respectively decreases (green line) or increases (red line) the amplitude of the contingent negative variation (CNV). Considering the “perception phase”, placebo treatments produce a decrease (blue line) in laser-evoked potential (LEP), an EEG wave that represents an early measure of nociceptive processes (**C**).

Considering the temporal aspects, pain can be studied during the expectation phase (e.g., when pain is anticipated) and during the perception phase (e.g., when pain is experienced) (Figure 2). During the expectation phase, activation of the anterior cingulate cortex, precentral and lateral prefrontal cortex, and periaqueductal gray has been documented; during the perception phase, deactivation has been found in different brain regions such as the mid- and posterior cingulate cortex, superior temporal and precentral gyri, the anterior and posterior insula, the claustrum and putamen, and the thalamus and caudate body [66] (Figure 2A). As for nocebo effects, where hyperalgesia is expected, increased activity in different brain regions involved in nociceptive processing and emotion regulation (such as the prefrontal cortex, anterior cingulate cortex and insula, primary somatosensory cortex, cerebellum, superior temporal gyrus, and operculum) has been documented [67,68,69,70]. During the perception phase, an enhanced activation has been found in regions such as the prefrontal cortex, anterior cingulate cortex, middle frontal gyrus, insula, claustrum, putamen, superior parietal lobule, amygdala, hippocampus, middle temporal gyrus, and periaqueductal gray [71,72] (Figure 2B). These findings concerning the temporal component of pain are confirmed by electroencephalographic (EEG) studies. Interestingly, placebos and nocebos can change EEG brain activity during both the expectation and perception phases [23,73,74]. For example, the expectation of receiving a nonpainful or painful stimulus respectively decreases or increases the amplitude of the contingent negative variation, i.e., an EEG slow negative wave that represents an objective measure of expectation of a specific incoming event (e.g., the expectation of analgesia or hyperalgesia) [23]. Considering the “perception phase”, placebo treatments produce decreased laser-evoked potentials, which represents an early measure of nociceptive processes, since it occurs 200–250 ms after painful stimulation [73]. The source of both these evoked potentials has been evaluated and the supplementary motor area, anterior cingulate cortex, middle cingulate cortex, and insula seem fundamental for contingent negative variation, and anterior cingulate cortex, operculum, and secondary sensorial cortex for laser-evoked potentials [75,76]. Moreover, placebo analgesia treatments significantly reduce the amplitude of the N1, P2, and P3 event-related potential components elicited by painful stimulation [77] (Figure 2C).

##### Central Nervous System

**Figure 3:**
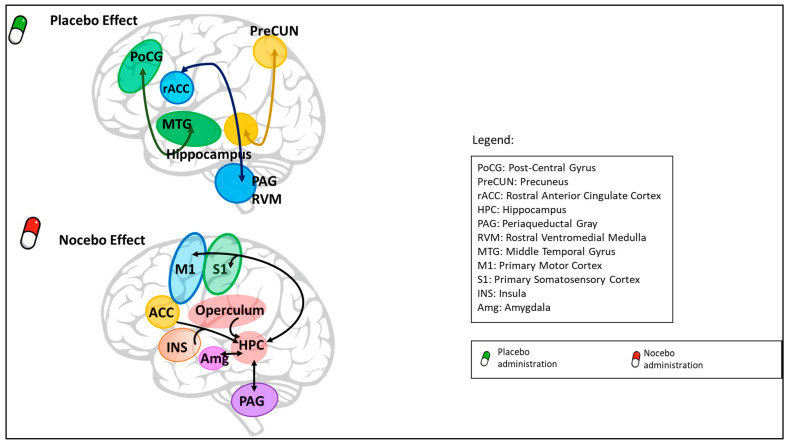
Connectivity analysis data. Connectivity studies have documented changes in functional connectivity in the placebo analgesic effect between PreCUN-HPC, MTG–PoCG, and rACC–PAG–RVM). In the nocebo hyperalgesic effect, functional connectivity changes have been observed among HPC/Operculum and many brain areas, namely ACC, INS, M1, and S1. In addition, functional connectivity between HPC and PAG and Amg has been suggested to play a role in the nocebo hyperalgesic effect.

Placebos and nocebos can affect the activity and the connectivity of cortical, subcortical, and spinal areas (Figure 3).

**High Central Nervous System Levels**

Starting from the cortical and subcortical levels in placebo expectation studies where inert treatments were delivered along with a verbal suggestion of symptom amelioration, an increase in μ opioid neurotransmission has been observed in different brain areas, such as the pre- and subgenual rostral anterior cingulate cortex [78,79,80,81,82], dorsolateral prefrontal cortex [79,80,81], orbitofrontal cortex [80,82], anterior insular cortex [79,80,81,82], nucleus accumbens [79,81,82], amygdala [79,80,82], thalamus [79,80], and periaqueductal gray [79,82]. On the contrary, when pain exacerbation is expected, a subjective increase in pain ratings has been reported along with increased activity in different brain regions involved in pain processing and emotion regulation, such as the prefrontal cortex, anterior cingulate cortex, and insula [70,71,83,84].

Similar results have been observed in open–hidden studies, where the open (placebo) condition, which maximizes the context effects, produced a behavioral analgesic effect along with deactivation of pain matrix areas, such as the mid and posterior cingulate cortex, insula, and thalamus, and activation of the dorsolateral prefrontal cortex and rostral anterior cingulate cortex [85]. On the contrary, in the hidden (nocebo) condition, which is a condition that significantly reduces the context effects, no changes in pain perception and no pain matrix deactivation were observed. Interestingly, expectations of drug interruption, e.g., expecting the analgesic effect to end, were followed by a blockage of drug analgesia and enhanced activity in the hippocampus [85].

Among all these areas, the dorsolateral prefrontal cortex and intraparietal sulcus seem to play a pivotal role in placebo responsiveness [31]. Studies supporting these conclusions are on healthy volunteers and patients with impairment in frontal regions. In Alzheimer’s patients who show compromised frontal lobes, the placebo analgesia negatively correlates with prefrontal activity impairment [86]. In healthy subjects, the prefrontal inactivation with repetitive transcranial magnetic stimulation results in a blockade of the placebo response [87], while active transcranial direct current stimulation, compared with sham transcranial direct current stimulation, boosts the placebo and blunts the nocebo effects [88]. Frontal activity seems to be crucial for placebo and nocebo responsiveness as researchers found a correlation between frontal activity and placebo effect magnitude; for example, placebo analgesia has been found to correlate with (1) fronto-parietal activity in regions associated with emotion regulation [63], (2) dorsolateral prefrontal cortex connectivity [89,90,91], and (3) opioid binding in the prefrontal cortex [45,65].

**Low Central Nervous System Levels**

Besides the study of high-level regions, recent studies have shown that placebo analgesia also involves nociception inhibition at the spinal level [92] and modulation of thalamocortical pathways related to nociception and pain [93,94]. At the same time, connectivity studies have documented changes in functional connectivity between precuneus-hippocampus and middle temporal gyrus-postcentral gyrus [95], and between the rostral anterior cingulate cortex and brain stem [63,90,96]. In particular, significant results suggest the involvement of the descending rostral anterior cingulate cortex-periaqueductal gray-rostral ventromedial medulla pain-modulating pathway, which in concert with other brainstem sites, such as the parabrachial nucleus, substantia nigra, and locus coeruleus, can influence the experience of pain by modulating activity at the level of the dorsal horn [97]. Interestingly, reductions in brain activity in areas that are not often considered, such as the habenula and the cerebellum, have been found [98]. Moreover, neural interactions between the prefrontal areas, brainstem, and spinal cord seem to regulate the nocebo effect. In particular, cognition interacts with the pain pathway through the rostral anterior cingulate cortex-periaqueductal gray-spinal axis, influencing nociceptive processing at the spinal level [99]. When nocebo hyperalgesia occurs, functional connectivity changes have been observed among hippocampus-operculum and other brain areas, including the anterior cingulate cortex, insula, primary motor cortex, and primary somatosensory cortex [71]. In addition, a recent study suggests a relevant role of the hippocampus and its functional connectivity with brain regions involved in the processing of sensory-discriminative aspects of pain, such as the periaqueductal gray and amygdala, in nocebo hyperalgesia [100].

Despite placebo analgesia and nocebo hyperalgesia interfering in pain perception and changing activity in different areas involved in nociceptive processing, it is still unclear if there is a strong correlation between the magnitude of the subjective placebo analgesia and objective changes in the latter areas. Given that the available literature suggests only a small subjective–objective correlation, it is likely that other mechanisms beyond the bottom-up nociceptive processing are involved in placebo analgesia [101]. Indeed, brain regions that are not associated with nociception but with self-regulation and high-level action selection, particularly the supplementary motor area, exhibit reduced activity during placebo analgesia. These effects may reflect a shift in motivation and decision making in the context of pain [31].

### 2.3. Genetics

Finally, a crucial and novel aspect of placebo and nocebo responsiveness is related to the role of genetic factors that can substantially contribute to these phenomena. The research in this field is in its early years, but it is plausible that placebo effects are determined by a complex network of genetic factors, individual medical experiences, and environmental factors [102]. The study of polymorphisms associated with placebo responsiveness has been focused on the systems involved in the placebo response, e.g., dopamine, opioid, and endocannabinoid systems [103,104,105]. For example, the polymorphism of the µ-opioid receptor gene (OPRM1) seems to be involved in the individual differences in placebo responsiveness [105,106]. Due to the high incidence of placebo effects in randomized controlled trials (RCTs) of treatments for mood diseases, an interplay with placebo-effect-related genes may also be present in the serotonergic system [103]. Several genes have been suggested to be involved in the serotoninergic system related to placebo remission [79,102]. Hall and colleagues coined the term “placebome” [103] to define the plausible genetic factors that influence the responsiveness to placebos [107]. The former created a placebome module consisting of 54 proteins and evaluated the proximity of the module to modules related to diseases or symptoms known to have a high or low-to-no placebo response by utilizing a seed connector algorithm. Results showed that the placebome was located proximate to the module for diseases or symptoms known to have a high placebo response and distal to conditions known to have a low-to-no placebo response [104]. It is worth noticing that, despite the role played by genetic factors in placebo responsiveness, results from a recent pilot twin study suggest that individual learning experiences are more important than genetic influences, at least in placebo analgesia induced through a conditioning paradigm [108].

### 2.4. Placebo and Nocebo Effects in Chronic Pain

The study of placebo and nocebo effects in chronic pain patients is extremely complicated. Patients with chronic pain are usually exposed to different long-lasting painful conditions, generally longer than three months, with different levels of pain experience [109]. Indeed, chronic pain is used as an “umbrella term” that incorporates a wide range of clinical conditions, ranging from fibromyalgia, migraine, musculoskeletal pain, or long-standing pain states with or without actual known causes [109]. Therefore, there are no consistent results for the occurrence and magnitude of placebo analgesia in chronic pain disorders [3]. Different studies report that placebo treatments successfully induce analgesia in chronic pain patients [90,110,111,112], and the effect seems to be stronger in women than in men [113]. RCTs point out that some of the common therapies for low back pain were no better than placebo [114] or only minimally better [115], suggesting that placebo responses can be large and clinically significant [116,117]. Other studies report mixed results. For example, in the meta-analysis of Morozov et al. (2022), placebo demonstrated a significant efficacy on subjective parameters (e.g., visual analogic scale and McGill pain questionnaire) [14]. Generally, a positive patient–clinician communication atmosphere seems a relevant aspect that triggers placebo analgesic effects; for instance, Kaptchuk et al. (2008) compared two placebo acupuncture treatments in patients with irritable bowel syndrome and showed that, while both treatments were superior to a natural history group, the positive therapeutic relationship further increased the efficacy of placebo acupuncture [111].

Overall, even if different studies have confirmed the occurrence of placebo analgesia in patients with chronic pain, it remains unclear if the mechanisms underlying these effects are different or similar to those observed in response to experimental pain protocols in healthy participants [13]. One crucial point is that, due to their personal medical experiences, both populations show completely different pain and treatment efficacy expectations [118,119]. These experiences would likely change the responsiveness to placebo or nocebo contexts. For example, the meta-analysis of Peerdman et al. (2016) indicates that expectations of patients may largely influence experimental and acute pain, whereas they have small effects on chronic pain [120]. Moreover, Muller et al. (2016) observed that, even if placebo analgesia was found to be large for both acute experimental and chronic pain, the two placebo responses were not related [118]. The main role of prior therapeutic experiences is supported by the results of Colloca et al. (2020) that showed a similar placebo analgesia magnitude in both healthy participants and chronic pain patients, which was directly linked to prior therapeutic experiences (conditioning procedure) [121].

Also, from a neurobiological point of view, there seem to be differences between patients and healthy controls in terms of placebo responsiveness, starting with the observation that naloxone appears not to block placebo analgesia in chronic pain states [110,122]. The results suggest that, in chronic pain patients, the opioid system may not be involved in placebo analgesia as in healthy subjects. From one perspective, it is surprising since pharmacological opioids are often used to treat chronic pain [123,124,125], but it is still true that the efficacy of opioids on chronic pain is debated, especially for long-term treatment [126]. A possible explanation for these results lays in the altered functioning of the opioid system as reported in chronic pain animal models [127] and human patients [128,129,130]. Different theories try to explain the persistence of pain in chronic conditions. For example, pain perception can be viewed as an inferential process in which top-down expectations and priors interact with bottom-up sensorial data. After administering a treatment, when bottom-up sensorial data changes, priors can be updated following bottom-up changes or maintained. In the case of chronic pain patients, there could be a bias in the interpretation of bottom-up information along with the use of immunization strategies that prevent the update of priors and expectations [131]. In line with this, chronic pain patients tend to explain ambiguous stimuli as pain- or condition-related without positively updating their previous expectations and cognitions [132,133,134,135]. An inability to update expectations based on outcomes (e.g., when the pain experience is less than anticipated) would result in a system that is poorly attuned to the external environment [135], and patients with chronic pain seem to lack this ability: studies show that patients are less capable of improving their performance on reward-dependent learning tasks [136,137,138,139] and showed an altered loss aversion in a monetary gambling task [140]. In line with this, it is suggested that the reward-related processes in the inability to update expectations are playing a role in the development of prolonged pain [141]. One hypothesis takes into account the possible absence of reward signaling related to endogenous opioid transmission [125], as supported by the studies on the altered opioid system in chronic pain patients [128,129,130].

Beyond the role of the opioid system, differences in the dopamine system, described both in animals and humans with chronic pain [142], may contribute to the development and maintenance of a chronic pain condition [143]. For example, a single-blinded-placebo trial in chronic pain patients showed that placebo responders had higher functional connectivity enriched by the dopamine transporter than nonresponders. This result suggests that those patients with the strongest dopamine-related neurotransmission might benefit the most from expectancy/placebo effects [125].

Differences in placebo responsiveness in chronic pain patients have also been related to other brain structures and function characteristics. In particular, (functional) Magnetic Resonance Imaging ((f)MRI) research demonstrated that subcortical limbic volume asymmetry, sensorimotor cortical thickness, and functional coupling of prefrontal regions, anterior cingulate cortex, and periaqueductal gray were predictive of placebo responses [90]. It is worth noting that these brain traits were present before administrating a placebo treatment, which provides evidence for a placebo responsiveness propensity and, as demonstrated using a machine learning algorithm, a biosignature to predict the placebo response at group level [90,144].

Despite these differences between healthy controls and chronic pain patients, close correspondence in mechanisms underlying placebo responses in these populations has also been found. For example, levels of activation in the dorsolateral prefrontal cortex and orbitofrontal cortex, as well as the coupling of the dorsolateral prefrontal cortex and rostral anterior cingulate cortex with antinociceptive circuitry [89,90], are believed to be part of both placebo responses [90].

Overall, it remains debatable whether the mechanisms underlying placebo responses in patients really differ from the ones in healthy controls, as well as whether there are true differences in these mechanisms in response to either acute or chronic pain. However, it seems plausible that the results of placebo research in experimental settings on healthy volunteers may not be totally transferable to placebo responses in chronic pain populations.

## 3. Future Directions for Clinical Practice

As documented in the previous section, the mechanistic placebo literature suggests that inert interventions provided within a specific context can relieve pain [5]. Translation of these findings into clinical practice requires the acknowledgement that positive clinical outcomes in patients seeking care for different painful conditions (e.g., musculoskeletal pain) are related to many factors [131]. Generally, an intervention’s effectiveness for a given patient may be attributable to a combined effect of: (1) factors such as natural history and regression to the mean: the natural history of many musculoskeletal disorders is favorable, and patients tend to seek care when their symptoms are at their worst, resulting in regression to the mean with repeated assessment over time; (2) the specific effects of the intervention resulting in improved outcomes regardless of the context of administration; and (3) factors related to the context of the intervention such as whether the patient expects the intervention to be effective and the relationship between the patient and the provider [145,146]. Positive and negative contexts influence the effectiveness of all pain management interventions [147,148,149]. For example, contextual effects accounted for more than 75% of the improvements observed in RCTs of interventions for osteoarthritis [150] and following surgical interventions for pain [151]. In patients with painful conditions, individual interventions often fail to show added value when directly compared to other interventions with modest treatment effects at best [152,153]. Observing only small differences in effects across multiple interventions that are different based on their theoretical working mechanisms suggests a significant role for contextual factors that these interventions have in common [131]. For instance, consciously seeking to maximize the contextual effects in clinical practice offers an intriguing opportunity to enhance treatment effects by maximizing the specific mechanisms of interventions as well as the context surrounding intervention administration [6,7].

Previously highlighted factors known to influence placebo analgesia also influence clinical outcomes in patients with different chronic pain conditions. For example, recovery expectations [154,155,156] and the relationship between the patient and provider [157] are known influential factors for the clinical outcome of patients experiencing musculoskeletal pain. Expectations mediating placebo analgesia appear to be depending on social learning [21,22,158,159,160]. Specifically, expectations may be formed and manipulated through verbal instruction, observation, and conditioning [158,160]. Experimental studies suggest that providing a placebo intervention with the following instruction: “the agent you have just received is known to powerfully reduce pain in some patients” [110], having a participant watch someone else experience pain relief in response to a placebo [161], or undergoing a conditioning protocol [162] are all approaches to enhance expectations which can result in increased placebo analgesia. Similar approaches in the clinic, such as educating patients on the effectiveness of a chosen intervention, making patients aware of the provider’s own personal observations of success, the use of patient testimonials, or providing interventions to which a patient has previously had positive experiences, may all be ways to maximize the contextual benefits of interventions for pain through the maximizing of expectations [62,120].

Therapeutic alliance is characterized in psychotherapy as the bond including trust and attachment between the patient and provider and includes consideration of agreement on the goals of therapy and assignment of tasks [163]. The literature on placebos suggests that therapeutic alliance can be enhanced and placebo analgesia increased when a sham intervention is administered by a provider who is warm and friendly, practices active listening, expresses empathy, and expresses confidence in the intervention [111,164,165]. These clinical results support the findings from the literature on experimental placebos [166]. Consequently, outcomes of patients presenting with pain may be improved when a strong therapeutic alliance is established between the patient and the provider [1,2].

In summary, patients with chronic pain may experience improved outcomes in response to an intervention for a variety of reasons beyond the specific effect of the intervention [6,7]. Contextual effects are a component of all interventions for pain that clinicians should implement in their clinical practice (Table 2). The literature on mechanistic placebos provides insight into how these effects can be successfully utilized in clinical practice.

## 4. Future Directions for Research and Clinical Trials

High-quality RCTs are the gold standard for treatment effectiveness. The traditional interpretation of null findings in placebo RCTs is considering the experimental intervention as ineffective. Specific to pain as an outcome, this assumption neglects the potential analgesic response to a placebo [145,146]. Consequently, a studied intervention providing no greater pain relief than a placebo comparator may suggest two equally effective interventions, potentially with differing mechanisms behind their effectiveness [167]. Different factors need to be considered for designing and interpreting placebo-controlled studies on interventions for pain [145,146]. The blinding of both patients and providers is an important consideration in placebo-controlled trials given that participants are made aware during the consent process of a 50% chance of receiving a placebo [146]. Blinding may be compromised due to poorly designed placebos which are not credible. Furthermore, blinding may be lost in placebo-controlled medication studies due to sensations unique to the studied intervention [168] or side effects in the active arm [169]. Based on a literature review of sham-controlled trials concerning back pain interventions, it appeared that a higher percentage of participants in active trial arms correctly identified their intervention, e.g., active and not sham, while blinding was successful in the sham arms of the studies [170]. Importantly, larger treatment effect sizes were observed in response to both the studied intervention and sham intervention when participants believed they received the active intervention [170]. Therefore, blinding should be carefully considered in placebo-controlled trials of pain management interventions and care should be taken to design sham or placebo comparators which are effective in maintaining blinding. Furthermore, blinding success should be assessed and reported in such trials [145,146].

Moreover, expectations are a primary mechanism of placebo analgesia [147]. Discrepancies between participant expectations concerning the success of a provided intervention between the active and placebo arms of a study could influence the observed outcomes [171]. Consequently, when designing placebo comparators for interventions for pain, care should be taken to assess expectations and ensure that the expectations for each arm of the study are similar [160].

Then, the true effect size of contextual effects on clinical outcomes requires additional consideration beyond the traditional two-arm placebo RCT. First, attributing changes in outcomes in a placebo treatment arm of a study to the placebo effect is temping; however, such an approach can be misleading [145,146]. Changes in the placebo arm should be considered as the placebo response; however, accurately measuring the placebo effect requires a no-treatment control group to account for influences such as natural history and regression to the mean [8].

Participants in an RCT are aware through the consent process of having a 50% chance of receiving a placebo. Consequently, individuals volunteering to participate in an RCT may differ from those presenting for clinical care, where expectations for improvement tend to be high [172,173]. Placebo mechanism studies differ from placebo-controlled studies given that participants are provided a placebo but instructed that they are receiving an effective intervention [147,148]. This study design is more consistent with clinical care in which interventions are generally provided by enthusiastic practitioners who instruct the patient of the likely effectiveness of the chosen intervention [147,148]. Placebo responses are greater in placebo mechanism studies than in placebo control studies [147] and similar approaches may result in a more accurate representation of the magnitude of contextual effect sizes in clinical practice. Furthermore, placebo-controlled studies may underestimate the effect of interventions. A literature review of studies on antidepressants observed significantly greater responses to treatment in terms of depression in studies with active comparators as compared to placebo-controlled studies [174]. Participants in studies with an active comparator were twice as likely to respond and one and a half times as likely to experience remission compared to participants in a traditional placebo-controlled study on antidepressants [174]. Such findings may be attributable to the expectations of participants in the active arm of the placebo-controlled studies who are also aware of the possibility that their intervention is a placebo [171]. Collectively, these findings suggest RCTs may underestimate both the placebo and treatment effects due to differences in expectations from those observed in clinical care [171]. Carefully designed studies may be necessary to account for the true magnitude of the influence of these factors on outcomes and provide a more accurate indication of their role in the effectiveness of interventions, offering opportunity for future research (Table 2).

## 5. Conclusions and Limitations

In summary, while studies on healthy participants seem consistent and provide a clear picture of how the brain reacts to different contexts at biological, neurophysiological, and genetical levels, there are no consistent results for the occurrence and magnitude of placebo and nocebo effects in chronic pain patients, mainly due to the heterogeneity of painful conditions. Thus, while it is a common experience that the same therapy offered in different contexts may influence the patient’s outcome in care settings representing an opportunity for clinicians, future studies on placebo and nocebo effects on patients with chronic pain are urgently needed, calling researchers and trialists to action worldwide.

This state-of-the-art paper presents some limitations. First, given that this paper comprises a narrative overview of the current state of the art, the included studies and data were not selected by adopting a systematic review approach. However, recommendations for performing a narrative biomedical review have been followed [10]. Second, the paper is mainly focused on the neurobiological and clinical aspects of placebo and nocebo effects, without describing the psychological mechanisms and determinants of these phenomena in detail. Third, the paper is limited to the specific topic of pain, even if it is well documented that there is not one sole placebo/nocebo effect, and instead many effects are mediated by a variety of psychological and biological mechanisms.

## Figures and Tables

**Table 1:** Narrative review methodology used for research and analysis [10].

Typology	Details
Sources accessed	*Database*: Cumulative Index to Nursing and Allied Health Literature—CINAHL, Excerpta Medica database—EMBASE, MEDLINE through PubMed, Web of Science. *Other*: bibliographic lists of relevant articles.
Search terms	*Key-words*: placebo, nocebo, effects, pain, acute, chronic, analgesia, hyperalgesia. *Boolean operators*: AND, OR.
Limits	*Time*: from inception of databases to 1st of January 2023. *Language*: English.
Studies included	*Design*: primary quantitative studies (e.g., experimental research, clinical trials) and secondary (e.g., narrative review, systematic review, metanalysis). *Target*: healthy participants, patients with acute and chronic pain of different origins (e.g., musculoskeletal, surgical). *Topic*: placebo and nocebo effects in acute and chronic pain.
Steps for writing	*Analysis*: collection, analysis, and organization of findings, grouping of findings with similar content. *Reporting*: organization of the main text into subsections, synthesis of findings into tables and figures, definition of key points for future research and practice, summary of new, evidence-based points.

Abbreviations: CINAHL, Cumulative Index to Nursing and Allied Health Literature; EMBASE, Excerpta Medica database.

**Table 2:** Key points for clinical practice and research.

Area	Actions
Clinical Practice	Considering the patient’s previous positive and negative experiences when drawing up the treatment plan. Evaluate the patient’s positive and negative expectations prior to the administration of therapy. Pay attention to the relationship and therapeutic alliance between the patient and provider during the care continuum. Emphasizing the clinical improvements that have occurred as a result of therapy. Consciously and conscientiously use contextual effects to enhance the specific effect of therapy.
Research	Ensuring the blinding of patients, evaluating and reporting it in placebo-controlled trials. Using comparators in sham groups that are similar in characteristics to the real treatments in placebo-controlled trials. Assess patient expectations in placebo-controlled trials. Recognize that a nontreatment control group to exclude confounders (e.g., the natural history of the disease) in placebo-controlled trials is necessary to establish the magnitude of the placebo effect size. Assess patient’s belief in having participated in the control or active group once placebo-controlled trials have ended.

## Data Availability

Being a state of the art review, this study does not contain original data.

## Abbreviations

RCT	Randomized Controlled Trial
fMRI	functional Magnetic Resonance Imaging
PET	Positron Emission Tomography
EEG	electroencephalography
VAS	Visual Analogue Scale
NRS	Numerical Rating Scale
CI	Confidence Interval
rTMS	repetitive Transcranial Magnetic Stimulation
ROI	Regions of Interest
M	Male
F	Female

## Appendix A

**Table A1:** Characteristics of the experimental placebo–nocebo studies included in this paper.

Paper ID	Sample Size (M, F, Not Analysed) *	Population Type	Pain Type/Pain Induction	Investigated Outcome	Outcome Measure	Level of Significance
Amanzio and Benedetti, 1999 [20]	229 (132, 97)	Healthy subjects	Experimental ischemic pain	Behavioral (Pharmacological)	Pain tolerance (min)	*p* < 0.05
Amanzio et al., 2001 [21]	364 (278 patients; 86 healthy controls)	Patients (thoracic surgery) and healthy controls	Postoperative pain; experimental ischemic arm pain	Behavioral (Pharmacological)	NRS (0–10)	*p* < 0.05
Benedetti et al., 1995 [40]	93 (52, 41)	Patients (thoracotomy for lung surgery)	Post-surgery pain	Behavioral (Pharmacological)	NRS (0–10)	*p* < 0.02
Benedetti et al., 1996 [39]	340 (154, 186)	Healthy subjects	Experimental ischemic pain	Behavioral (Pharmacological)	NRS (0–10)	*p* < 0.05
Benedetti et al., 1997 [38]	180 (119, 61)	Patients (video-assisted thoracoscopy)	Post-surgery pain	Behavioral (Pharmacological)	NRS (0–10)	*p* < 0.05
Benedetti et al., 2006 [42]	49 (23, 26)	Healthy subjects	Experimental ischemic pain	Behavioral (Pharmacological)	NRS (0–10)	*p* < 0.05
Benedetti et al., 2006 [86]	44 (28 patients (11, 17), 16 controls)	Patients (Alzheimer’s disease) and healthy subjects	Burning pain after venipuncture	Electrophysiological (EEG)	NRS (0–10)	*p* < 0.05
Benedetti et al., 2010 [41]	40 (20, 20)	Healthy subjects	Experimental ischemic pain	Behavioral (Pharmacological)	Tolerance time	*p* < 0.05
Benedetti et al., 2011 [36]	82 (41, 41)	Healthy subjects	Experimental ischemic pain	Behavioral (Pharmacological)	Tolerance time	95%CI
Benedetti et al., 2014 [54]	74 (30, 44)	Healthy subjects	Hypobaric hypoxia headache	Behavioral (Pharmacological)	NRS (0–10)	95%CI
Benedetti et al., 2022 [37]	149 (82, 67)	Healthy subjects	Experimental ischemic pain	Behavioral (Pharmacological)	0–10 rating scale	*p* < 0.05
Bingel et al. 2011 [85]	22 (15, 7)	Healthy subjects	Heat pain	Neuroimaging (fMRI)	VAS (0–100)	*p* < 0.05
Bingel et al., 2022 [100]	22 (15, 7)	Healthy subjects	Heat pain	Neuroimaging; functional connectivity (fMRI)	VAS (0–100)	*p* < 0.05
Bush et al., 2021 [95]	37 (12, 25)	Healthy subjects	Heat pain	Neuroimaging; functional connectivity (fMRI)	VAS (0–100)	*p* < 0.05
Camerone et al., 2021 [16]	166 (78, 88, 9)	Healthy subjects	Electrical stimuli	Behavioral	NRS (0–10)	*p* < 0.05
Camerone et al., 2021 [17]	77 (24, 24, 29)	Healthy subjects	Cold pressor test (CPT)	Behavioral	Numerical Pain Intensity (0–100)	*p* < 0.05
Camerone et al., 2022 [18]	51 (24, 27, 10)	Healthy subjects	Cold pressor test (CPT)	Behavioral	NRS (0–10)	*p* < 0.05
Carlino et al., 2015 [73]	34 (20, 14)	Healthy subjects	Laser stimulation	Electrophysiology (EEG)	NRS (0–10)	*p* < 0.05
Carlino et al., 2016 [26]	80 (34, 46)	Healthy subjects	Electrical stimuli	Behavioral	NRS (0–10)	*p* < 0.05
Colloca et al., 2006 [24]	30 (5, 25)	Healthy subjects	Electrical stimuli	Behavioral	NRS (0–10)	*p* < 0.05
Colloca et al., 2008 [21]	116 (0, 116)	Healthy subjects	Electrical stimuli	Behavioral	NRS (0–10)	*p* < 0.05
Colloca et al., 2010 [25]	46 (16, 30)	Healthy subjects	Electrical stimuli	Behavioral	VAS (0–10	*p* < 0.05
Colloca et al., 2016 [50]	109 (55, 54, 1)	Healthy subjects	Electrical stimuli	Behavioral	VAS (0–10)	*p* < 0.05
Colloca et al., 2019 [105]	160 (58, 102)	Healthy subjects	Electrical and heat stimuli	DNA genotyping; epistasis	VAS (0–10)	*p* < 0.001
Colloca et al., 2020 [121]	763 (363 patients (85, 278); 400 healthy controls (162; 238)	Patients (chronic orofacial pain) and healthy subjects	Heat stimuli	Behavioral	VAS	*p* < 0.05
Disley et al., 2021 [30]	104 (10, 65, 29)	Healthy subjects	Cold pressor test (CPT)	Behavioral	VAS (0–100)	*p* = 0.05
Eippert et al., 2009 [34]	48 (48, -, 8)	Healthy subjects	Heat pain	Neuroimaging (fMRI)	VAS (0–100)	*p* ≤ 0.05
Eippert et al., 2009 [92]	15 (15, 0)	Healthy subjects	Heat pain	Neuroimaging (fMRI)	VAS (0–100)	*p* < 0.05
Ellerbrock et al., 2015 [35]	40 (20, 20, 1)	Healthy subjects	Heat pain	Neuroimaging; functional connectivity (fMRI)	VAS (0–100)	*p* < 0.05
Fuentes et al., 2014 [164]	117	Patients (chronic low back pain)	-	Behavioral	NRS (0–10)	*p* < 0.05
Hashmi et al., 2014 [91]	42	Patients (chronic knee osteoarthritis)	Heat pain	Neuroimaging (fMRI)	Gracely Sensory Scale (0–20)	*p* < 0.05
Jarcho et al., 2016 [46]	15 (0, 15)	Healthy subjects	Heat pain	Neuroimaging (PET; fMRI)	VAS (0–100)	*p* < 0.005
Kaptchuk et al., 2008 [111]	262 (63, 199)	Patients (irritable bowel syndrome)	-	Behavioral	Global improvement scale (range 1–7); adequate relief of symptoms; symptom severity	*p* < 0.01
Kelley et al., 2009 [165]	189	Patients (irritable bowel syndrome)	-	Behavioral	Combined outcome (IBS Symptom Severity Scale; IBS Quality of Like Scale; IBS Global Improvement Scale; IBS Adequate Relief)	*p* < 0.05
Kessner et al., 2013 [51]	80 (80, 0)	Healthy subjects	Heat pain	Behavioral (Pharmacological)	Visual Analogue Scale (0–100)	*p* < 0.05
Klinger et al., 2017 [112]	48 (12, 36)	Patients (chronic back pain)	Electrical stimuli	Behavioral	NRS (0–10)	95% CI
Kong et al., 2006 [57]	24 (13, 11)	Healthy subjects	Heat pain	Neuroimaging (fMRI)	0–20 Sensory Box Scale	*p* < 0.0001 for ROI *p* = 0.05
Kong et al., 2008 [71]	20 (5, 8, 7)	Healthy subjects	Heat pain	Neuroimaging (fMRI)	Gracely Sensory and Affective Scales	*p* < 0.05
Koyama et al., 2005 [70]	10 (8, 2)	Healthy subjects	Heat pain	Neuroimaging (fMRI)	VAS	*p* < 0.01
Krummenacher et al., 2010 [87]	40 (40, 0)	Healthy subjects	Heat pain	rTMS	VAS (0–10)	*p* ≤ 0.05
Kube et al., 2020 [29]	117 (48, 53, 16)	Healthy subjects	Heat pain	Behavioral	Pain tolerance	*p* < 0.05
Lieberman et al., 2004 [58]	52 (29 active drug; 23 placebo condition)	Patients (irritable bowel syndrome)	-	Neuroimaging (PET)	Symptom diary (4 weeks)	*p* < 0.005
Malfiet et al., 2019 [79]	83	Patients (chronic neck pain)	-	Behavioral	VAS (0–100)	*p* = 0.05
Martins et al., 2022 [125]	56	Patients (chronic knee osteoarthritis)	-	Neuroimaging; functional connectivity (fMRI)	VAS (0–10)	*p* < 0.05
Morton et al., 2010 [74]	67 (21, 35, 11)	Healthy subjects	Laser stimulation	Electrophysiological (EEG)	0–10 scale	*p* = 0.05
Müller et al., 2016 [118]	50 (27, 32, 1)	Patients (chronic pain)	Pressure-pain stimuli	Behavioral	VAS (0–100)	*p* < 0.05
Olson et al., 2021 [113]	280 (65, 215)	Patients (chronic orofacial pain)	Heat pain	Behavioral	VAS (0–100)	*p* < 0.05
Peciña et al., 2015 [106]	50 (21, 29)	Healthy subjects	5% hypertonic saline	DNA genotyping; Neuroimaging (PET)	VAS (0–100)	*p* < 0.05
Petrovic et al., 2002 [60]	9	Healthy subjects	Heat stimuli	Neuroimaging (PET)	VAS (0–100)	*p* = 0.005
Petrovic et al., 2010 [59]	24 (9, 15)	Healthy subjects	Heat stimuli	Neuroimaging (PET; fMRI)	VAS (0–100)	*p* < 0.05
Piedimonte et al., 2017 [23]	34(16, 18, -)	Healthy subject	Electrical stimuli	Electrophysiological (EEG)	NRS (0–10)	*p* < 0.05
Ploghaus et al., 1999 [67]	12 (7, 5)	Healthy subjects	Heat stimuli	Neuroimaging (fMRI)	VAS (0–10)	*p* < 0.05
Pollo et al., 2001 [81]	38	Patients (thoracotomized patients)	-	Behavioral	NRS (0–10)	*p* < 0.01
Porro et al., 2002 [69]	30 (10, 16, 4)	Healthy subjects	Acid solution injection	Neuroimaging (fMRI)	0–100 scale rating	*p* < 0.05
Price et al., 1999 [162]	40 (16, 24)	Healthy subjects	Heat pain	Behavioral	VAS (0–10)	*p* < 0.05
Price et al., 2007 [61]	9	Patients (irritable bowel syndrome)	Barostat balloon distension—pressure stimuli	Neuroimaging (fMRI)	100-unit rating scale	*p* < 0.05
Prossin et al., 2022 [55]	37 (12, 25)	Healthy subjects	Hypertonic saline injection	Neuroimaging (PET, MRI)	VAS (0–100)	*p* < 0.05
Rief et al., 2012 [168]	144 (50, 904)	Healthy participants	Heat pain	Behavioral	Pain threshold change in °C	*p* < 0.05
Ruscheweyh et al., 2014 [98]	60 (30 patients, 30 controls)	Patients (cerebellum infarction) and healthy subjects	Heat; pressure; pinprick pain	Behavioral	NRS (0–10)	*p* < 0.05
Sawamoto et al., 2000 [83]	10 (10, 0)	Healthy subjects	Laser thermal stimulation	Neuroimaging (fMRI)	0–100 scale	*p* < 0.05
Schmid et al., 2015 [84]	44 (22, 22)	Healthy subjects	Rectal distension	Neuroimaging (fMRI)	VAS (0–100)	*p* < 0.05
Schwartz et al., 2022 [161]	44 (18, 26)	Patients (chronic low back pain)	-	Behavioral	NRS (0–10)	*p* < 0.05
Scott et al., 2007 [49]	48 (30 Study1; 16 Study2; 18 Male controls)	Healthy subjects	5% hypertonic saline injection	Neuroimaging (Study1—PET, fMRI Study2—fMRI)	VAS (0–100)	*p* < 0.05
Scott et al., 2008 [43]	20 (9, 11); 18 (18, 0)	Healthy subjects	Sustained muscle pain challenge	Neuroimaging (PET, MRI)	VAS (0–100)	*p* < 0.0001 for ROI *p* = 0.05
Skyt et al., 2018 [47]	19 (10, 9)	Patients (neuropathic pain)	Pinprick-evoked pain; wind-up-like pain	Behavioral	VAS (0–10; 0–100)	*p* < 0.05
Tétreault et al., 2016 [89]	98 (17 Study1; 39 Study2; 42 Study3)	Patients (chronic knee osteoarthritis pain)	-	Neuroimaging (fMRI)	VAS (0–10); Western Ontario and McMaster Universities Osteoarthritis Index	*p* < 0.05
Tinnermann et al., 2017 [99]	57 (27, 22, 8)	Healthy subjects	Heat stimuli	Neuroimaging (fMRI)	VAS (0–100)	*p* < 0.05
Tu et al., 2021 [88]	81 (44, 37)	Healthy subjects	Heat stimuli	Neuroimaging (fMRI); tDCS	Gracely Sensory Scale (0–20)	*p* < 0.05
Vachon-Presseau et al., 2018 [90]	129 (43 placebo group, 20 controls, 66 excluded)	Patients (chronic back pain)	Back pain intensity	Neuroimaging (MRI, fMRI)	VAS (0–10)	*p* < 0.05
Vachon-Presseau et al., 2022 [144]	181 (94 randomized to 3 arms, 87 excluded)	Patients (chronic low back pain)	Back pain intensity	Neuroimaging (fMRI)	Likert Scale (twice a day)	*p* < 0.05
Van der Meulen et al., 2017 [72]	30 (13, 17)	Healthy subjects	Heat stimuli	Neuroimaging (fMRI)	VAS (0–100)	*p* < 0.05
Vase et al., 2003 [82]	13	Patients (irritable bowel syndrome)	Evoked rectal distension; heat pain	Behavioral	VAS (0–10)	*p* < 0.05
Vase et al., 2005 [110]	26 (0, 26)	Patients (irritable bowel syndrome)	Rectal distension	Behavioral (Pharmacological)	VAS (0–10)	*p* < 0.05
Vecchio et al., 2021 [77]	63 (31, 32)	Healthy subjects	Electrical stimuli	Electrophysiological (EEG)	7 point Likert scale	*p* = 0.05
Wager et al., 2004 [64]	47	Healthy subjects	Shock pain; heat pain	Neuroimaging (fMRI)	10 point scale	*p* < 0.05
Wager et al., 2007 [65]	15 (15, 0)	Healthy subjects	Heat stimuli	Neuroimaging (PET)	VAS (0–10)	*p* < 0.05
Wager et al., 2011 [63]	47	Healthy subjects	Shock pain; heat pain	Neuroimaging (fMRI)	10 point scale	*p* < 0.001
Wanigasekera et al., 2018 [96]	16	Patients (Post-traumatic neuropathic pain)	-	Neuroimaging (MRI)	NRS (0–10)	*p* = 0.05
Weimer et al., 2019 [108]	39 (25 monozygotic; 14 dizygotic twin pairs)	Healthy subjects	Heat pain	Behavioral	NRS (0–10)	*p* < 0.05
Wrobel et al., 2014 [48]	50 (28, 32, 12)	Healthy subjects	Heat pain	Neuroimaging (fMRI); Pharmacological	VAS (0–100)	*p* < 0.05

* If not differently specified.
